# Genome sequence analysis of La Crosse virus and *in vitro *and *in vivo *phenotypes

**DOI:** 10.1186/1743-422X-4-41

**Published:** 2007-05-08

**Authors:** Richard S Bennett, David R Ton, Christopher T Hanson, Brian R Murphy, Stephen S Whitehead

**Affiliations:** 1 Laboratory of Infectious Diseases, National Institute of Allergy and Infectious Diseases, National Institutes of Health, Bethesda, MD 20892, USA

## Abstract

**Background:**

La Crosse virus (LACV), family *Bunyaviridae*, is a mosquito-borne virus recognized as a major cause of pediatric encephalitis in North America with 70–130 symptomatic cases each year. The virus was first identified as a human pathogen in 1960 after its isolation from a 4 year-old girl who suffered encephalitis and died in La Crosse, Wisconsin. The majority of LACV infections are mild and never reported, however, serologic studies estimate infection rates of 10–30/100,000 in endemic areas.

**Results:**

In the present study, sequence analysis of the complete LACV genomes of low-passage LACV/human/1960, LACV/mosquito/1978, and LACV/human/1978 strains and of biologically cloned derivatives of each strain, indicates that circulating LACVs are genetically stable over time and geographic distance with 99.6–100%, 98.9–100%, 97.8–99.6%, and 99.2–99.7% amino acid identity for N, NsS, M polyprotein, and L proteins respectively. We identified 5 amino acid differences in the RNA polymerase and 4 nucleotide differences in the non-coding region of the L segment specific to the human virus isolates, which may result in altered disease outcomes.

**Conclusion:**

All three wild type viruses had similar *in vitro *growth kinetics and phenotypes in mosquito C6/36 and Vero cells, and similar levels of neurovirulence and neuroinvasiveness in Swiss Webster mice. The biologically cloned derivative of LACV/human/1960 was significantly less neuroinvasive than its uncloned parent and differed in sequence at one amino acid position in the G_N _glycoprotein, identifying this residue as an attenuating mutation.

## Background

La Crosse virus (LACV), family *Bunyaviridae*, is a mosquito-borne pathogen endemic in the United States. LACV infection results in 70–130 clinical cases a year and is the major cause of pediatric arboviral encephalitis in North America [1-3]. LACV was first identified as human pathogen in 1960 after its isolation from a 4 year-old girl from Minnesota who suffered meningoencephalitis and later died in La Crosse, Wisconsin [4,5]. The majority of LACV infections are mild and never reported, however serologic studies estimate annual infection rates of 10–30/100,000 in endemic areas [2,3,6,7]. LACV is a member of the California serogroup of viruses in the genus *Orthobunyavirus*. The serogroup contains members found on five continents that include human pathogens such as La Crosse, Snowshoe hare, and Jamestown Canyon viruses in North America; Guaroa virus in North and South America; Inkoo and Tahyna viruses in Europe; and Lumbo virus in Africa [8]. Children who recover from severe La Crosse encephalitis may have significantly lower IQ scores than expected and a high prevalence (60% of those tested) of attention-deficit-hyperactivity disorder [2]. Seizure disorders are also common in survivors [9]. LACV can also cause encephalitis in immunosuppressed adults [10]. Projected lifelong economic costs associated with neurologic sequelae range from $48,775–3,090,398 per case [11]. At present, a vaccine or FDA approved antiviral therapy is not available.

LACV maintains an enzootic life cycle with the hardwood forest dwelling, tree-hole mosquito, *Aedes triseriatus*, which lives in the eastern half of the United States breeding in tree holes and outdoor containers [12]. *Ae. triseriatus *mosquitoes feed on Eastern gray squirrels (*Sciurus carolinensis*) and Eastern chipmunks (*Tamias striatus griseus*) which serve as amplifying hosts for LACV, and undergo sub-clinical infections while maintaining serum viremias high enough to infect feeding mosquitoes [13,14]. Interestingly, the virus can be maintained in the mosquito population in the absence of vertebrate hosts by transovarial (vertical) transmission, thus allowing the virus to over-winter in mosquito eggs. Mosquito infection is lifelong and mosquitoes can become dually infected with other bunyaviruses allowing for the development of intra-genus reassortants [15-18].

LACV virions are pleomorphic (90–100 nm in diameter) and have a lipid envelope containing the heteromultimer glycoprotein [19]. The genome consists of three single-stranded, negative-sense RNA genome segments designated small (S), medium (M), and large (L). Each genome segment is complexed with the nucleoprotein (N) to form three separate nucleocapsids. The termini of the 3' and 5' non-coding regions (NCR) of each segment are complementary and highly conserved. The S segment encodes two proteins in overlapping reading frames: the nucleoprotein (N) and a non-structural protein (NS_S_). In the related Bunyamwera virus, NS_S _inhibits transcription via blocking host cell RNA polymerase II which decreases overall host cell protein synthesis in mammalian cells including a decrease in both the induction of interferon and its signaling in infected cells [20,21]. Recombinant LACV virions lacking the NS_S _gene are viable, indicating that the NS_S _is a nonessential accessory protein [22]. The M segment encodes a single polyprotein (M polyprotein) that is post-translationally processed into two glycoproteins (G_N _and G_C_) that form a heteromultimer in the virion and a non-structural protein (NS_M_) of unknown function [23]. The L segment encodes a single open reading frame for the RNA dependent RNA polymerase (L) [24,25]. The L polymerase uses host-cell 5' mRNA sequences, including the cap structures, to prime its own mRNA synthesis, a process that also contributes to the observed shut-off of host cell protein synthesis following infection.

To identify a nucleotide sequence of LACV associated with the wild type phenotype, i.e. replication competent in insect and mammalian cells and able to cause encephalitic disease in suckling and weanling mice by a peripheral and intracerebral route of inoculation, we sequenced the complete genomes of three low-passage LACV isolates, namely, LACV/human/1960, LACV/human/1978, LACV/mosquito/1978, isolated over a 18 year period of time. Biologically cloned derivatives of each virus were also sequenced. The level of neurovirulence and neuroinvasiveness for each of the three virus isolates and their cloned derivatives was determined in mice by assessing clinical disease following intracerebral or intraperitoneal administration of virus. LACV strains appear highly genetically stable in nature, grow to high titers in monkey and mosquito cell cultures, and are highly neurovirulent and neruoinvasive for mice even at low dosage. Since one of the long-term goals of this project is to develop a live attenuated virus vaccine for LACV, the identification of a nucleotide sequence of LACV that specifies a wild type phenotype was seen as an essential first step in this process. In this study, we have also identified a single amino acid substitution in G_N _in one of the cloned LACV strains that greatly decreases LACV neuroinvasiveness. Such a mutation may be useful in developing live-attenuated virus vaccine candidates.

## Results

### Sequence analysis of viral genomes

LACV genomes were sequenced for two reasons. First, we wanted to determine the genetic diversity of LACV isolated in different regions of the United States at different times, and second, we sought to define a complete genomic sequence that is associated with the wild type phenotype of virulence in mice by both peripheral and intracerebral routes of inoculation. The sequence of only two complete LACV genomes was previously reported, one human isolate (LACV/human/1978, GenBank accession numbers NC_004108–NC_004110) and one mosquito isolate (strain 77, LACV/mosquito/1977, GenBank accession numbers DQ196118–DQ196120) (Table 1). We sequenced two additional isolates, including the original La Crosse, Wisconsin virus (LACV/human/1960) and the LACV/mosquito/1978 virus (Table 1), and re-sequenced LACV/human/1978 after an additional passage in tissue culture to confirm its identity prior to further biological characterization. In addition, LACV/human/1960, LACV/mosquito/1978, and LACV/human/1978 parental wild-type viruses were biologically cloned to generate a genetically homogeneous viral preparation, and the full sequence of these cloned preparations was also determined. Thus, we have generated full-length sequence for 3 pairs of cloned and uncloned LACV strains. For LACV/human/1978, the newly derived sequence (EF485033-35) was used for all subsequent comparisons since several differences with the former sequence were identified. An examination of the virulence phenotype of these three parental and cloned viruses in mice should define one or more full-length sequences that have a wild type phenotype.

**Table 1:** Passage history and geographic location of isolation/infection of the LACV isolates for which complete genomic sequences are available.

**Virus**	**Location**	**Passage history**^**a**^	**GenBank Accession number**
LACV/human/1960	Minnesota	C6/36 2	EF485030–EF485032
LACV/human/1960-clone	Minnesota	C6/36 2, Vero 4	NA^b^
LACV/mosquito/1978	North Carolina	Mouse brain 1, Vero 3	EF485036–EF485038
LACV/mosquito/1978-clone	North Carolina	Mouse brain 1, Vero 7	NA
LACV/human/1978	Wisconsin	Mouse brain 1, BHK 2, Vero 1	EF485033–EF485035
LACV/human/1978-clone	Wisconsin	Mouse brain 1, BHK 2, Vero 5	NA
LACV/mosquito/1977	Wisconsin	Unknown	DQ196118–DQ196120^c^
LACV/human/1978	Wisconsin	Mouse brain 1, BHK 2	NC_004108–NC_004110^d^

^a^Cell/tissue type followed by number of passages.

^b^Sequence not submitted. Genetic comparisons with uncloned parental wild-type stocks found in Table 3.

^c^Previous submission by Cheng et al. 2005.

^d^Previous submission by Hughes et al. 2002. Sequence from a derivative of this virus (one additional passage in Vero cells) was generated for this study (EF485033–EF485035) and used for subsequent comparisons since several differences with this sequence were identified.

A comparison of the complete genomic nucleotide sequences from low passage LACV isolates of either human or mosquito origin isolated over an 18 year period of time from two geographically different regions of the United States (Table 2) indicated little sequence divergence. The S, M, and L genome segments for each virus were 984, 4526, and 6980 nucleotides in length, respectively. The nucleotide length of the segments and the encoded open reading frames for each of the isolates are identical. The S, M, and L segments from each virus isolate share a high nucleotide sequence identity ranging from 97.9–100%, 95.7–99.8%, and 95.7–99.4% respectively (Table 2). The N, NSs, M polyprotein, and L protein open reading frames are 235, 92, 1441, and 2263 amino acid codons in length, respectively. The percent identity for encoded proteins is also highly conserved among the isolates with 99.6–100%, 98.9–100%, 97.8–99.6%, and 99.2–99.7% identity for the N, NSs, M polyprotein, and L proteins, respectively (Table 2). Although the process of biological cloning resulted in an additional four passages in Vero cells, these passages had a minimal effect on genetic stability. A maximum of four nucleotide changes were observed between a wild type parental virus stock and its biologically cloned derivative, and no more then one amino acid change was observed in any cloned virus (Table 3). This level of sequence divergence between parental and cloned virus is much less than that between LACV isolates (> 500 nucleotides differences for human/1978 compared to mosquito/1978), clearly identifying each isolate as a separate strain.

**Table 2:** Nucleotide and amino acid identity (%) of the LACV genomic segments and their predicted protein products.

		Human/1960	Human/1978	Mosquito/1978	Mosquito/1977	
	
**S segment/N protein**						
Human/1960		-	99.6	100	100	Amino Acid
Human/1978		99.8	-	99.6	99.6	
Mosquito/1978		98.1	97.9	-	100	
Mosquito/1977	Nucleotide	100	99.8	98.1	-	

**S segment/NSs protein**						

Human/1960		-	98.9	100	100	Amino acid
Human/1978		99.8	-	98.9	98.9	
Mosquito/1978		98.1	97.9	-	100	
Mosquito/1977	Nucleotide	100	99.8	98.1	-	

**M segment/M polyprotein**						

Human/1960		-	99.4	97.8	99.6	Amino acid
Human/1978		99.6	-	97.8	99.4	
Mosquito/1978		95.8	95.8	-	97.7	
Mosquito/1977	Nucleotide	99.8	99.5	95.7	-	

**L segment/L protein**						

Human/1960		-	99.7	99.2	99.5	Amino acid
Human/1978		99.4	-	99.2	99.5	
Mosquito/1978		95.9	95.7	-	99.5	
Mosquito/1977	Nucleotide	97.1	96.9	96.0	-	

**Table 3:** Nucleotide differences between wild type parental and biologically cloned virus.

**LACV Virus**	**Nucleotide substitution in indicated LACV segment**^**a**^		
	
	**S**	**M**	**L**
		
human/1960-clone	A525T	A503G^b^	A1837G
		C2221T	
human/1978-clone	No changes^c^	T391C	No changes
		A1636G	
		A1929G^b^	
mosquito/1978-clone	A719G^b^	No changes	A31G
			G33A

^a^Parental nucleotide on left, nucleotide substitution in the cloned virus on right.

^b^Indicates a nucleotide substitution resulting in an amino acid substitution.

^c^Parental and cloned virus have identical sequences.

**Figure 1:**
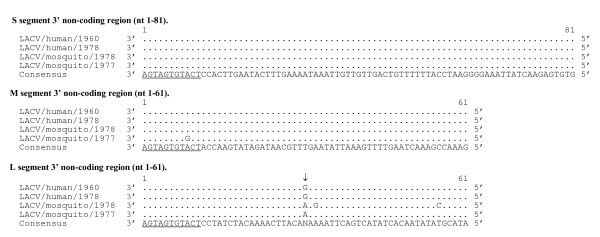
**Alignment of 3' non-coding region of S, M, and L genome segments (cDNA presented)**. S segment 3' NCR shows highly conserved sequence with no nucleotide changes from the consensus. For each segment the consensus sequence consists of three or more sequences sharing the same nucleotide at a given position and areas with no clear consensus are indicated with an "N". A single nucleotide change was reported in the LACV/mosquito/1977 published sequence at position 9 of the M segment. For the 3' NCR of the L segment, 2 changes from the consensus were observed in LACV/mosquito/1978 with position 31 having no clear consensus. Underlined sequence indicates region conserved among all three segments. Putative host-specific nucleotide sequences are indicated with an arrow (↓).

**Figure 2:**
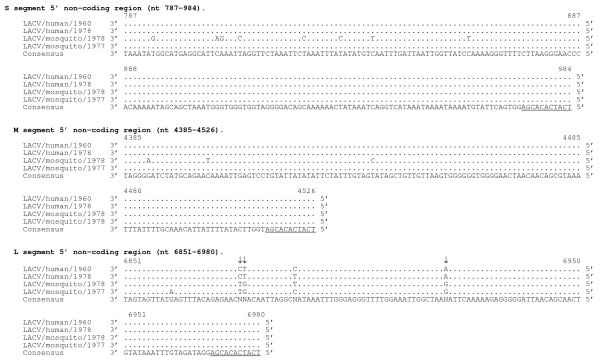
**Alignment of 5' non-coding region of S, M, and L genome segments (cDNA presented)**. Among the two human isolates only one nucleotide difference was observed in the NCR of the L segment at position 6888. For each segment, the consensus sequence consists of three or more sequences sharing the same nucleotide at a given position and areas with no clear consensus are indicated with and "N". Underlined sequence indicates region conserved among all three segments. Putative host-specific nucleotide sequences are indicated with an arrow (↓).

The sequences of two LACV isolates from humans were compared with two isolates from mosquitoes to identify amino acids that are shared by LACVs of human origin but that differ from LACVs of mosquito origin. Such sequences differences are referred to as host-specific sequence substitutions. Five such host-specific amino acid substitutions were identified, and all were located in the L protein (Table 4). Four nucleotide substitutions in the non-coding region (NCR) of the L segment at nucleotides 31, 6876, 6877, and 6921 also appear to be host-specific (Figure 1 and 2).

**Table 4:** Host specific amino acid differences are located in the RNA polymerase (L).

**Amino acid position**	**Amino acid residue**	
	
	**Human**	**Mosquito**
129	V	I
484	K	R
1040	E	G
1713	T	A
1906	A	S

Genetic comparisons used all wild-type parental sequences generated for this paper and published sequences of mosquito strain LACV-77 (DQ196118–DQ196120). RNA polymerase amino acid 922 was R or G in the human isolates and K in the two mosquito isolates.

The 3' and 5' genome ends of the LACV genomes were also highly conserved. The first 11 and last 11 nucleotides were identical for each segment end (Figure 1 and 2). Each 3' NCR was identical for the S (nt 1–81) and only one nucleotide differed from the consensus in the M (nt 1–61) segments. The 3' NCR of L (nt 1–61) from LACV/mosquito/1978 differed from the consensus by 2 nucleotides (Figure 1). The 5' NCR of LACV/mosquito/1978 differed from the S, M, and L consensus by 8, 3, and 1 nucleotides, respectively. For the L segment, a clear consensus sequence was not identified at position 31 of the 3' end and at nucleotide positions 6876, 6877, 6888, and 6921 in the 5' NCR. Between the two human isolates, one nucleotide difference in the NCR was identified at position 6888 of the 5' NCR of L (Figure 2).

### In vitro growth kinetics

**Figure 3:**
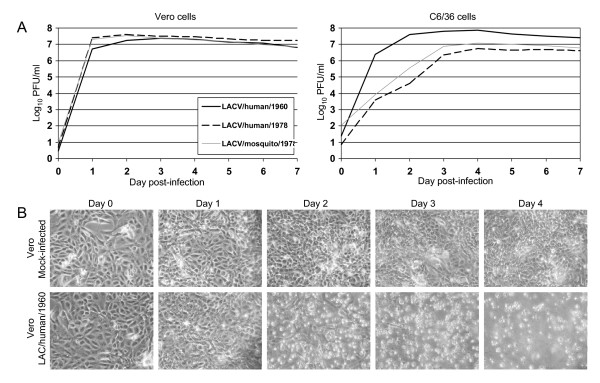
**Growth kinetics and CPE of LACV strains**. **A**. Growth kinetics of LACV/human/1960, LACV/human/1978, and LACV/mosquito/1978 in Vero cells or C6/36 cells infected at an MOI of 0.01. **B**. Photographs of mock or LACV/human/1960 infected Vero cell monolayers from panel "A". Cell rounding and detachment from the flask can be seen on days 2–4 post-infection in infected monolayers.

Comparison of *in vitro *growth of LACV/human/1960, LACV/mosquito/1978 and LACV/human/1978 viruses was performed in Vero cells and C6/36 cells (Figure 3). All viruses replicated to high titers in both cell types. LACV/human/1960 replicated more quickly in C6/36 cells, possibly as a result of originally being isolated in these cells. Growth kinetics in Vero cells for all three viruses were nearly identical reaching approximately 10^7 ^PFU/ml in 24 hours (Figure 3A). Each of the three viruses replicated efficiently in C6/36 cells, but, in contrast to the rapid development of cytopathic effects (CPE) in Vero cells, infection of C6/36 cells was not cytopathic over the seven day period (data not shown). CPE associated with LACV infection of Vero cells consisted of cell rounding and detachment from the flask with 80% of the monolayer destroyed by 3 days post-infection (Figure 3B).

### LACV clinical disease in mice: LD_50_

All six LACV isolates were neurovirulent in Swiss Webster mice regardless of previous passage in mouse brains. In suckling or weanling mice, the LD_50 _values ranged from -0.50 to 1.50 log_10 _PFU (Table 5). Thus, each of the three genome sequences of the parental and cloned viruses is a sequence of a fully neurovirulent virus. Of the six LACV isolates tested, five were neuroinvasive for mice of both ages whereas the LACV/human/1960-clone did not induce clinical disease in weanling mice even after inoculation of 10^6 ^PFU (Table 5). It was determined that this virus has a nucleotide substitution (A503G) resulting in a single amino acid change at position 148 (Threonine→Alanine) in the G_N _(formerly G2) attachment glycoprotein and three silent nucleotide substitutions (Table 3). This suggests that the alanine residue at position 148 attenuates neuroinvasiveness. Clinical disease in mice included lethargy, tremors, seizures, and limb paralysis, although there was no consistent sequence to the progression of disease.

**Table 5:** La Crosse neurovirulence and neuroinvasiveness after intracerebral (IC) or intraperitoneal (IP) inoculation of Swiss Webster mice.

**Virus**	**Neurovirulence (IC)** (LD_50 _log_10 _PFU)		**Neuroinvasiveness (IP)** (LD_50 _log_10 _PFU)	
		
	**Suckling mice**^**a**^	**Weanling mice**^**b**^	**Suckling mice**	**Weanling mice**
LACV/human/1960	1.35	1.30	2.17	2.56
LACV/human/1960-clone^c^	1.37	-0.25	1.76	> 6.0
LACV/human/1978	0.37	-0.50	0.57	1.75
LACV/human1978-clone	0.42	-0.15	0.83	1.25
LACV/mosquito/1978	1.19	1.13	1.08	1.84
LACV/mosquito/1978-clone	1.36	1.50	1.29	2.40

^a^Suckling mice are 2–3 days old.

^b^Weanling mice are 21–23 days old.

^c^Biologically cloned viruses were obtained by terminally diluting wild type parental virus three times, then amplified by an additional passage in tissue culture.

## Discussion

As an initial step in vaccine development, we defined a panel of LACV genomic sequences that is associated with wild type *in vitro *and *in vivo *phenotypes. This was done by examining the phenotypic properties of parental and biologically cloned derivatives of three LACV viruses. For our purpose, a LACV was defined as exhibiting a wild type phenotype if it was fully replication competent in insect and mammalian cells and was able to cause encephalitic disease in suckling and weanling mice by a peripheral and intracerebral route of inoculation. Although LACV does not appear virulent in either mosquitoes or in its amplifying hosts in nature, it is clearly virulent in humans and mice resulting in severe central nervous system (CNS) infections in both species. Therefore, this virulence for CNS of mice is the phenotype that we would like to modify as a surrogate phenotype for the development of a attenuated vaccine candidates for humans. Five of the six LACV isolates studied, three parental and two cloned viruses, had the wild type virulence phenotype *in vivo*. These defined wild type sequences can now be used as a baseline for the identification of mutations that attenuate LACV for the CNS. A live, attenuated LACV virus vaccine could reduce the occurrence of LACV encephalitis in the U.S., and possibly could be useful as a genetic background for the creation of chimeric vaccines against other pathogens in the *Bunyaviridae *family as has been successfully done for the flaviviruses and paramyxoviruses [26,27].

In the present study, the complete genomic sequence of two wild type LACV isolates was determined, and one previously determined sequence was confirmed with some minor clarifications of the published sequences. These three LACV sequences, along with a previously determined sequence, were compared to examine the extent of genetic diversity of the LACV genome. Although these four viruses originated in distinct geographic locations and were isolated from either humans or mosquitoes over a period of 18 years, the viruses exhibited a remarkable level of genetic relatedness independent of passage history, location of isolation, or host. The N open reading frame of the S segment was the most conserved protein sequence (≥ 98% identity) among the isolates, followed by that of the RNA polymerase L, NSs, and M polyprotein (≥ 95% identity). It is possible that ecological factors, such as the need to replicate efficiently in both mammalian and insect hosts, have selected for a genotype that has obtained maximum fitness in both hosts. In such a model, variants that arise by genetic drift may be quickly selected against by either host. Clearly, a greater number of isolates from both hosts still need to be examined, but recovery of LACV from humans is unusual and isolates are rare.

Since we had complete sequences for two viruses isolated from mosquitoes and two from humans, it was possible to search for host specific sequences that distinguish between LACV isolates obtained from the two species. Five amino acid substitutions were found in the RNA polymerase and may define the host-specific genetic differences. In addition, four nucleotide differences in the NCR of the L segment also appear to be host-specific. Such host-specific differences were not identified in the S or M segments. None of the differences is located in the conserved *Bunaviridae *L protein motifs A-D [28]. Since the human isolates would only be obtained from symptomatic cases, it is possible that the L segment of the LACV might be a determinant of virulence in humans and that only those LACVs with a specific L segment sequence are isolated from humans with disease. Since there were nine host-specific differences between the human and mosquito isolates, it is unlikely, although not impossible, that nine shared changes would have co-developed during the replication of a LACV in two different humans following infection with the mosquito genotype. Rather, it is likely that there are subsets of LACV strains in nature, only some of which might be capable of causing severe disease in humans. Since virus with mosquito- or human-specific L segment sequences did not differ in virulence in mice, this suggestion of an association of a sequence with human disease is offered with great caution. As additional virus isolates from humans become available for sequence analysis, it will be important to monitor these specific amino acids and nucleotides for their association with human disease. In addition, it will be interesting to determine if viruses with the human host specific sequences can be directly isolated from mosquitoes.

As a first step towards vaccine development, we sought to establish a reproducible murine model of LACV infection suitable for pathogenesis and vaccine safety/efficacy studies. In humans, disease incidence is age dependent with the majority of cases in children under 15 years old[13]. Previous studies in BALB/C mice using LACV/human/1960, passaged nine times in suckling mouse brain and two times in BHK cells, resulted in an age-specific decrease in neuroinvasiveness most notable at 3 weeks of age [29]. In our Swiss Webster mouse model, the LD_50 _values were similar for both age groups, with the exception of the biologically cloned LACV/human/1960. This virus was neuroinvasive in suckling mice but not in weanling mice presumably due to a mutation in the G_N _glycoprotein. Although the G_N _glycoprotein is believed to play a role in binding of LACV virions to mosquito midgets, it may also have a role in the development of CNS disease[30]. Future work will focus on understanding which step in disease progression is blocked after infection with a virus bearing this mutation in G_N_. The use of 3-week-old weanling mice was advantageous because they are more mobile than suckling mice allowing for a more detailed observation of clinical disease manifestations. Following inoculation of 5-week-old Swiss Webster mice with 10^5 ^PFU of LACV/mosquito/1978 (a dose 100% lethal for 3-week-old mice) only 50% (3 of 6) became ill (data not shown), suggesting that Swiss Webster mice will also be useful in understanding age-dependent neuroinvasiveness of LACV.

## Conclusion

Taken together, these results have implications for our future vaccine development efforts. First, LACV is genetically stable over time and distance, suggesting that a vaccine based on any of these virus isolates should induce a protective immune response against most, if not all, circulating LACV strains. Second, we have identified a mutation in the G_N _glycoprotein that appears to be associated with decreased neuroinvasiveness, yet does not affect virus replication in tissue culture. Clearly, our current *in vivo *testing allows for the identification of mutations effecting neuroinvasiveness. Third, we have identified a convenient mouse model that will allow us to screen numerous mutant viruses for attenuated neuroinvasiveness/neurovirulence and allow us to continue to evaluate the pathogenesis of LACV infection and disease.

## Methods

### Cells and viruses

C6/36 cells (*Aedes albopictus *mosquito larvae) were maintained in Earle's MEM supplemented with 10% fetal bovine serum (HyClone), 2 mM L-glutamine (Invitrogen), and 1 mM non-essential amino acids. Vero cells (African green monkey kidney) were maintained in OptiPRO™ SFM medium (Invitrogen) supplemented with 4 mM L-glutamine (Invitrogen).

LACV/human/1960 was isolated from post-mortem brain tissue collected from a Minnesota patient hospitalized in La Crosse, Wisconsin and passaged two times in C6/36 cells. LACV/mosquito/1978 was isolated from mosquitoes collected in North Carolina and passaged once in mouse brain and three times in Vero cells. LACV/human/1978 was isolated from post-mortem brain tissue collected in Wisconsin and passaged once in mouse brain, twice in BHK-21 cells, and once in Vero cells (Table 1).

### Isolation of biologically cloned viruses

Biological clones were generated by terminal dilution in Vero cell cultures. Virus stocks were serially diluted in 2-fold increments and inoculated onto 90% confluent monolayers of Vero cells in 96-well plates using eight wells per dilution. After five days of incubation, cell culture fluid was removed to a holding plate, and the cell monolayers were fixed and stained for 10 minutes with crystal violet solution (1% crystal violet in equal volumes of ethanol and methanol). The virus was selected as a clonal derivative when only 1 or 2 of the 8 wells in a single row was positive for LACV CPE. Each virus was terminally diluted three times (sequentially), amplified in Vero cell culture, and subjected to genome sequence analysis.

### Virus titrations

Vero cells in 24-well plates were infected in duplicate with ten-fold serial dilutions of LACV and overlayed with OptiMEM (Invitrogen) supplemented with 1% methylcellulose, 5% FBS, 2.5 μg/ml amphotericin B, and 20 μg/ml ciprofloxicin. Five days after infection the overlay was removed and cells were washed twice with PBS. The cells were fixed and stained for 10 minutes with crystal violet solution, viral plaques were identified by characteristic CPE, and titers are expressed as log_10 _PFU/ml.

### Sequence analysis of viral genomes

Viral RNA was isolated using either QIAamp Viral RNA kit (Qiagen) or EZ1 Viral RNA mini kit (Qiagen). Reverse transcription (RT) was performed using random hexamer primers and SuperScript™ First-Strand Synthesis System for RT-PCR (Invitrogen). Overlapping PCR fragments were generated using LACV specific primers and Advantage cDNA polymerase reaction kit (BD Biosciences Clontech). PCR fragments of up to 2000 bp were purified and both strands directly sequenced using viral specific primers in BigDye-terminator cycle sequencing reactions analyzed on an ABI3730 genetic analyzer (Applied Biosystems). Sequence fragments were assembled into a consensus sequence using AutoAssembler 2.1 software (Applied Biosystems).

To sequence the 5' and 3' genome ends, viral RNA was isolated using QIAamp Viral RNA kit (Qiagen) from virus infected cells at 24–48 hours post infection for the 3' non-coding region (NCR) or from cell culture supernatant fluid for the 5' NCR. Viral RNA was reverse transcribed using Reverse Transcriptor (Roche) at 55°C with random hexamer primers for the 3' NCR or at 60–70°C with genome specific primers that enhanced reverse transcription though RNA secondary structures. cDNA was purified with High Pure (Roche) and a poly-A tail was added to the 3' end of the cDNA using 5'/3' RACE Kit, Second Generation (Roche). Genome ends were then amplified using virus and poly-A specific primers. Purified PCR fragments were sequenced as described above.

### *In vitro *growth kinetics

LACV/human/1960, LACV/mosquito/1978, and LACV/human/1978 were used to infect 95% confluent monolayers of C6/36 or Vero cells at a multiplicity of infection of 0.01 and incubated for one hour to allow attachment. Infected monolayers were washed twice with sterile PBS and overlaid with medium. Tissue culture fluid (0.5 ml) was collected every 24 hours after infection, mixed 1:10 with 10× SPG buffer (final concentration 218 mM sucrose, 6 mM L-glutamic acid, 3.8 mM dibasic potassium phosphate, pH 7.2), and frozen. Daily samples were titrated as described above. Cell monolayers were photographed on day 0, 1, 2, 3, and 4 for LACV/human/1960 infected, or non-infected Vero cells.

### LACV clinical disease in mice

The lethal dose_50 _(LD_50_) of LACV virus was evaluated in Swiss Webster suckling and weanling mice (Taconic Farms, Germantown, NY). All animal experiments were carried out in accordance with the regulations and guidelines of the National Institutes of Health. Litters of 3 day-old suckling mice (n > 8/dose) were inoculated with serial dilutions of wild type or biologically-cloned LACV in a volume of 10 μl intracerebrally (IC) or 100 μl intraperitoneally (IP). The experiment was repeated with 3 week-old weanling mice (n = 6/dose), however, the older mice were anesthetized with isofluorane prior to IC inoculation. All mice were carefully observed twice daily for clinical disease including tremors and limb paralysis. Because clinically moribund mice were humanely euthanized before succumbing to infection, moribundity served as a surrogate for the determination of lethality.

## Competing interests

The author(s) declare that they have no competing interests.

## Authors' contributions

RSB performed animal studies, sequence analysis and drafted the manuscript. DRT performed *in vitro *growth analysis and participated in animal studies. CTH conducted sequencing. BRM and SSW supervised the study and participated in its design and planning. All authors read and approved the final manuscript.
